# Computational Studies on Water-Catalyzed Mechanisms for Stereoinversion of Glutarimide Intermediates Formed from Glutamic Acid Residues in Aqueous Phase

**DOI:** 10.3390/ijms20102410

**Published:** 2019-05-15

**Authors:** Tomoki Nakayoshi, Shuichi Fukuyoshi, Koichi Kato, Eiji Kurimoto, Akifumi Oda

**Affiliations:** 1 Graduate School of Pharmacy, Meijo University, 150 Yagotoyama, Tempaku-ku, Nagoya, Aichi 468-8503, Japan; 184331503@ccmailg.meijo-u.ac.jp (T.N.); kato-k@kinjo-u.ac.jp (K.K.); kurimoto@meijo-u.ac.jp (E.K.); 2 Institute of Medical, Pharmaceutical and Health Sciences, Kanazawa University, Kakuma-machi, Kanazawa, Ishikawa 920-1192, Japan; fukuyosi@p.kanazawa-u.ac.jp; 3 Department of Pharmacy, Kinjo Gakuin University, 2–1723 Omori, Moriyama-ku, Nagoya, Aichi 463-8521, Japan; 4 Institute for Protein Research, Osaka University, 3–2 Yamadaoka, Suita, Osaka 565-0871, Japan

**Keywords:** d-amino acid residues, non-enzymatic reaction, stereoinversion, density functional theory

## Abstract

Aspartic acid (Asp) residues are prone to non-enzymatic stereoinversion, and Asp-residue stereoinversion is believed to be mediated via a succinimide (SI) intermediate. The stereoinverted Asp residues are believed to cause several age-related diseases. However, in peptides and proteins, few studies have reported the stereoinversion of glutamic acid (Glu) residues whose structures are similar to that of Asp. We previously presumed that Glu-residue stereoinversion proceeds via a glutarimide (GI) intermediate and showed that the calculated activation barriers of SI- and GI-intermediate stereoinversion are almost equivalent in the gas phase. In this study, we investigated the stereoinversion pathways of the l-GI intermediate in the aqueous phase using B3LYP density functional methods. The calculated activation barrier of l-GI-intermediate stereoinversion in the aqueous phase was approximately 36 kcal·mol^−1^, which was much higher than that in the gas phase. Additionally, as this activation barrier exceeded that of Asp-residue stereoinversion, it is presumed that Glu-residue stereoinversion has a lower probability of proceeding under physiological conditions than Asp-residue stereoinversion.

## 1. Introduction

**Scheme 1:**
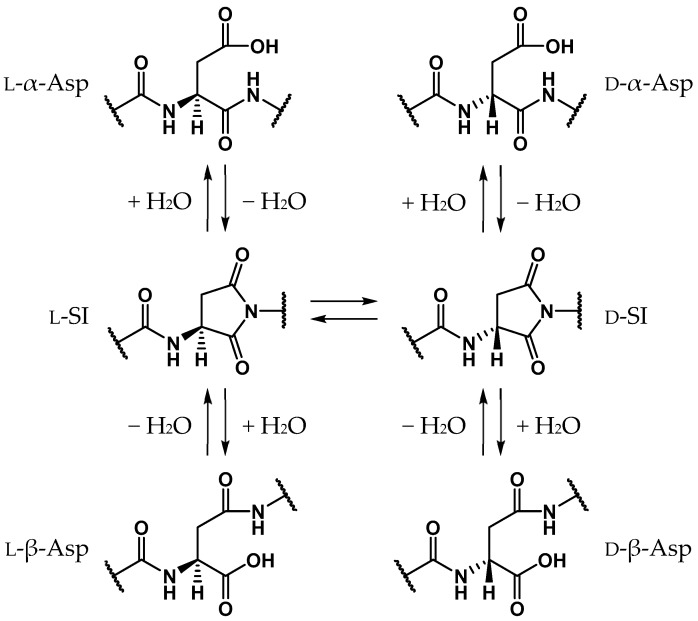
SI-mediated stereoinversion and/or isomerization mechanism of Asp residues.

**Scheme 2:**
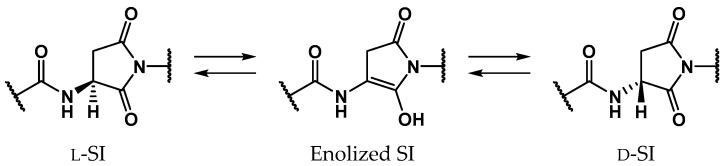
SI-intermediate stereoinversion mechanism via enolized SI intermediates.

Proteins comprise approximately 20 amino acids, which, except for glycine, have asymmetric carbon atoms. For a long time it was believed that human proteins comprised only l-amino acids, and that d-amino acid residues are not contained in living bodies [1,2,3,4,5,6,7,8]. Recently, however, d-amino acid residues have been detected in biological proteins in various aging tissues, e.g., crystallin in the lens [9,10,11,12], amyloid β in the brain [5,8,13], and elastin in the aorta, ligaments, and skin [14,15,16]. As these proteins are biosynthesized from only l-amino acids, d-amino acid residues identified in aging proteins are assumed to be formed by the stereoinversion of l-amino acid residues. The formation of d-amino acid residues dramatically affects the surrounding environment and the stereoinversion of certain amino acid residues produces significant conformational changes in the entire protein [17,18,19]. Additionally, the detected amounts of d-amino acid residues were found to increase with aging, and it is considered that the stereoinversion of l-amino acid residues can cause several age-related diseases such as cataracts [9,10,11,12] and Alzheimer’s disease [5,8,13]. Until now, most of the d-amino acid residues detected in aging tissues were aspartic acids (Asp). Asp-residue stereoinversion and isomerization is believed to non-enzymatically proceed via the formation of a five-membered ring succinimide (SI) intermediate. Subsequently, three types of isomerized Asp residues are formed: l-β-, d-α-, and d-β-Asp residues (Scheme 1) [1,2]. The SI intermediates are formed by the nucleophilic attack of the main-chain amide nitrogen of the adjacent residue on the C-terminal side, i.e., the (*n* + 1) residue, on the carboxyl carbon of the Asp-residue side chain. As the carbanion formed by the α-proton (H_α_) abstraction of the SI intermediate is stabilized by the resonance effect, the SI intermediate may be more prone to stereoinversion than other amino acids [20]. Following the enolization of the SI intermediate, the α-carbon is converted from a sp^3^- to a sp^2^-hybridized state, and the asymmetric carbon vanishes. Thus, along with the reketonization of enolized SI intermediates, not only l-SI but also d-SI intermediates are formed (Scheme 2). Kuge et al. experimentally estimated that the activation energies of the Asp-residue stereoinversion were 25.7–29.0 kcal·mol^−1^ in distilled water using synthesized elastin fragment peptides [21]. Although Asp-residue stereoinversion proceeds non-enzymatically, computational studies have suggested that some small molecule catalysts are required for this reaction [22,23]. Takahashi et al. suggested that Asp-residue stereoinversion can be catalyzed by water molecules, which are abundantly present in living bodies, using density functional theory (DFT) calculations [23,24,25,26,27]. In the previous study by Takahashi et al., the activation energy of SI-intermediate stereoinversion catalyzed by two water molecules was estimated as 28.2 kcal·mol^−1^ [24], and this was consistent with the experimental results.

**Scheme 3:**
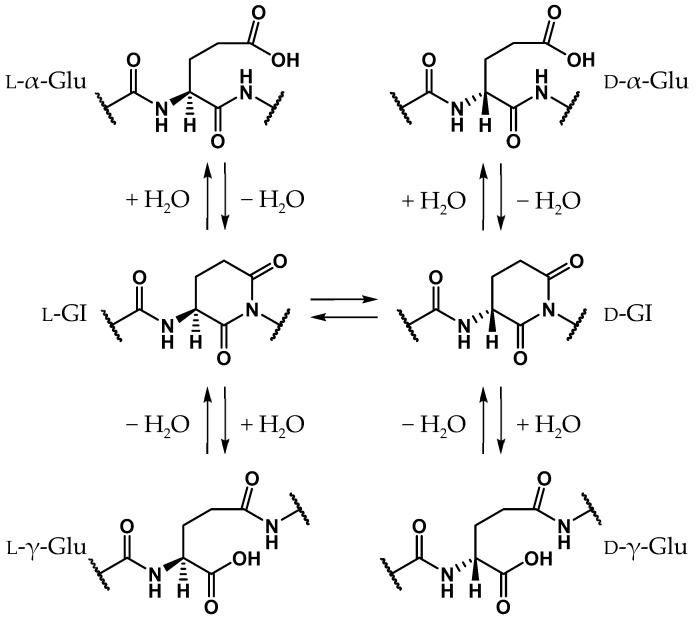
Proposed GI-mediated stereoinversion and/or isomerization mechanism for Glu residues based on that of Asp residues.

**Scheme 4:**
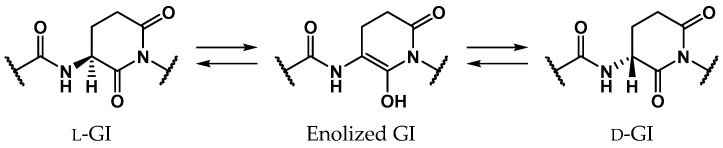
Proposed GI-intermediate stereoinversion mechanism via enolized GI intermediates

Glutamic acid (Glu) is a proteinogenic amino acid, and its chemical structure resembles that of Asp. Although stereoinversion and isomerization of Asp residues were detected in various aging tissues as mentioned above, few reports mention detection of stereoinverted and isomerized Glu residues in human proteins [12]. Studies on the stereoinversion of amino acid residues in proteins have only just begun, and experimental techniques for analyzing d-amino acid residues in proteins are not yet fully developed. Thus, it is not yet clear why detected amounts of stereoinverted and isomerized Glu residues are extremely small in comparison with Asp. To investigate why stereoinversion of Glu residues is less frequently observed compared to that of Asp residues, we previously studied the Glu-residue stereoinversion mechanism using quantum chemical calculations [28,29,30]. In previous studies, it was hypothesized that Glu-residue stereoinversion proceeded via the same pathway as that of the Asp-residue; the amide nitrogen of the (*n* + 1) residue nucleophilically attacks the Glu-residue side-chain carboxyl carbon to form a six-membered ring glutarimide (GI) intermediate, which converts to stereoinverted and/or isomerized Glu residues (Scheme 3) [28]. In a reported pathway [28,29,30], stereoinversion of l-GI intermediates was proposed to proceed via enolized GI intermediates (Scheme 4). The calculated activation energy of two-water-catalyzed GI-intermediate stereoinversion was estimated as 28.1 kcal·mol^−1^, and there was no substantial difference between the calculated activation barriers for SI-intermediate and GI-intermediate stereoinversion. However, in these studies, all calculations were performed in the gas phase, and hydration effects were not included. The cytoplasmic proteins are exposed to water. As the solvent affects the chemical properties of solute molecules, such as conformation and reaction rate [31,32], it appears important to include solvent effects in calculating the chemical reactions of amino acid residues. Therefore, in this paper, we have reported the GI-intermediate stereoinversion mechanism in the aqueous phase, employing the polarizable continuum model (PCM).

## 2. Results

**Figure 1:**
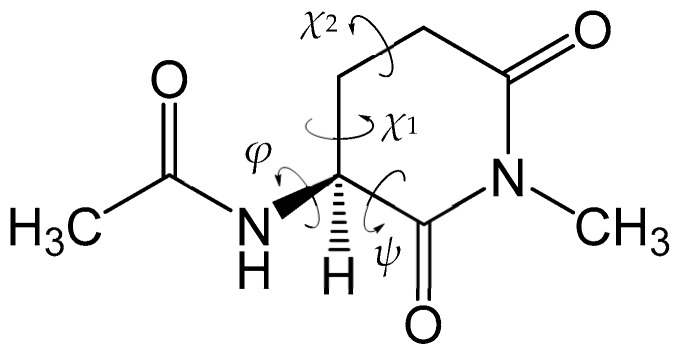
Chemical structure of the model compound (Ace‒l-GI‒Me) used in this study. The dihedral angles *φ* (C‒N‒C_α_‒C) and *ψ* (N‒C_α_‒C‒N), which characterize the main-chain conformation, and the dihedral angles *χ*_1_ (N‒C_α_‒C_β_‒C_γ_) and *χ*_2_ (C_α_‒C_β_‒C_γ_‒C_δ_), which characterize the side-chain conformation, are indicated.

Figure 1 shows the model compound in the present study, in which an l-GI residue is capped with acetyl (Ace) and methyl (Me) groups on the N- and C-termini, respectively. This model compound (Ace‒l-GI‒Me) was also used in our previous studies [28]. To avoid confusion, the backbones corresponding to the main and side chains of the Glu residue are defined as the main and side chains of the l-GI residue, respectively. The dihedral angles *φ* (C‒N‒C_α_‒C) and *ψ* (N‒C_α_‒C‒N) characterize the main-chain conformation, and the dihedral angles *χ*_1_ (N‒C_α_‒C_β_‒C_γ_) and *χ*_2_ (C_α_‒C_β_‒C_γ_‒C_δ_) characterize the side-chain conformation. Additionally, two water molecules were placed around the model compound, and we investigated the two-water-catalyzed stereoinversion mechanism of l-GI residues in the aqueous phase employing the PCM. All the energy minima and transition state (TS) geometries were optimized without any constraints by the DFT calculations using the B3LYP/6-31+G(d,p) level of theory. All the relative energies of the energy minima and TS geometries were corrected for the zero-point energies (ZPEs) obtained by vibrational frequency calculations.

First, we attempted to obtain the TS connecting the l-GI and enolized GI residues in the aqueous phase using the conformation and configuration of a reactant complex determined by our previous study [28]; however, this ended in failure. Therefore, we explored a novel configuration of catalytic water molecules for the l-GI-residue stereoinversion, and the four pathways described below were identified. In all the pathways, one water molecule, W1, abstracted the H_α_, and enolization proceeded by triple proton transfer mediated by two water molecules, W1 and W2. That is, two water molecules, W1 and W2, placed around the model compound acted as catalysts for the enolization of l-GI residues.

### 2.1. Pathway 1

**Figure 2:**
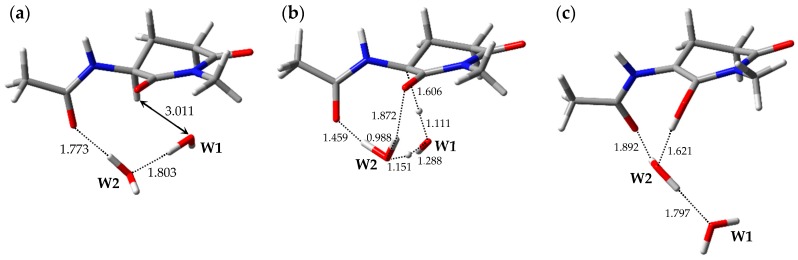
Optimized geometries of the enolization system in Pathway 1: (**a**) 1-RC (*φ* = −101°, *ψ* = −159°, *χ*_1_ = −179°, *χ*_2_ = −53°); (**b**) 1-TS (*φ* = −88°, *ψ* = −152°, *χ*_1_ = 177°, *χ*_2_ = −48°); and (**c**) 1-PC (*φ* = −100°, *ψ* = −171°, *χ*_1_ = −158°, *χ*_2_ = −46°). The selected interatomic distances are shown in Å.

The optimized geometries of the enolization system in Pathway 1, i.e., reactant complex 1-RC, TS 1-TS, and product complex 1-PC, are shown in Figure 2. In the Supplementary Materials, Tables S1–S3 present the Cartesian coordinates of 1-RC, 1-TS, and 1-PC. 1-RC is a complex which is composed of a capped l-GI residue and two water molecules, W1 and W2. In 1-RC, W1 was located 3.011 Å away from H_α_ of the l-GI residue. W2 included two hydrogen bonds, one with W1 and the other with the carbonyl oxygen of the (*n* − 1) residue. Their hydrogen bond lengths were 1.803 and 1.773 Å, respectively. Enolization of the l-GI residue was mediated by triple proton transfer among l-GI and two water molecules, from the C_α_ of the l-GI residue to W1, from W1 to W2, and from W2 to the main-chain carbonyl oxygen of the l-GI residue. Figure S1 shows the vibrational mode corresponding to the imaginary frequency (i.e., transition vector) of 1-TS in the Supplementary Materials, and the triple proton transfer appears to occur along the transition vector. During enolization, W2 was rotated, and a new hydrogen bond was formed between W2 and the main-chain carbonyl oxygen of the l-GI residue. 1-TS is a TS connecting two energy minima, 1-RC and 1-PC, and vibrational frequency calculations estimated that 1-TS has a single imaginary frequency of 975*i* cm^−1^. In 1-TS, the C_α_‒H_α_ bond length was increased to 1.606 Å. Complex 1-PC was formed from 1-TS by triple proton transfer via two water molecules. When 1-PC was formed from 1-TS, significant W1 migration and W2 rotation occurred. Complex 1-PC comprised a capped enolized GI residue and two water molecules. In 1-PC, W2 formed three hydrogen bonds with W1, the carbonyl carbon oxygen of the (*n* − 1) residue, and the OH proton of the enol moiety; these hydrogen bond lengths were 1.797, 1.892, and 1.621 Å, respectively. During enolization from 1-RC to 1-PC, all the defined dihedral angles were altered by less than 30°, and both the GI-residue main- and side-chain conformations were not significantly changed. The relative energies of 1-TS and 1-PC with respect to 1-RC were 35.1 and 20.3 kcal·mol^−1^, respectively.

### 2.2. Pathway 2

**Figure 3:**
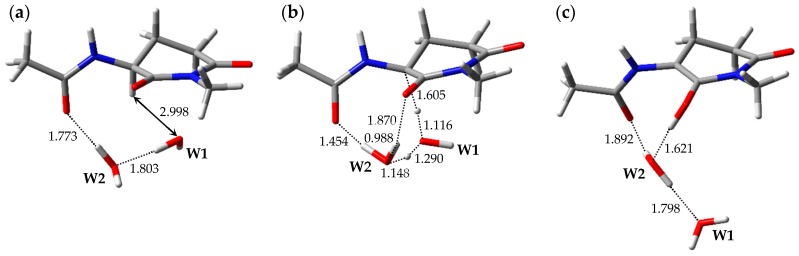
Optimized geometries of the enolization system in Pathway 2: (**a**) 2-RC (*φ* = −101°, *ψ* = −158°, *χ*_1_ = −180°, *χ*_2_ = −53°); (**b**) 2-TS (*φ* = −87°, *ψ* = −152°, *χ*_1_ = 176°, *χ*_2_ = −48°); and (**c**) 2-PC (*φ* = −100°, *ψ* = −171°, *χ*_1_ = −158°, *χ*_2_ = −46°). The selected interatomic distances are shown in Å.

The optimized geometries of the enolization system in Pathway 2, i.e., reactant complex 2-RC, TS 2-TS, and product complex 2-PC, are shown in Figure 3. In the Supplementary Materials, Tables S4–S6 present the Cartesian coordinates of 2-RC, 2-TS, and 2-PC. 2-RC is a complex which is composed of a capped l-GI residue and two water molecules, W1 and W2, and the geometry of 2-RC closely resembled that of 1-RC. The distance between W1 and H_α_ of the l-GI residue was 2.998 Å. W2 interacted with W1 and with the carbonyl oxygen of the (*n* − 1) residue, and these hydrogen bond lengths were 1.803 and 1.773 Å, respectively. Enolization of the l-GI residue was mediated by triple proton transfer among l-GI and two water molecules. As enolization progressed, W2 rotated significantly. 2-TS is a TS connecting two energy minima, 2-RC and 2-PC, and included an eight-membered ring “interaction bridge” connecting the C_α_ and main-chain carbonyl oxygen of the l-GI residue, and triple proton transfer proceeded along these interactions. Figure S2 shows the transition vector of 2-TS in the Supplementary Materials. 2-TS was estimated to have a single imaginary frequency of 991*i* cm^−1^ by vibrational frequency calculations. Although 2-TS has a geometry closely resembling that of 1-TS, the W1 orientations in these were significantly different. Complex 2-PC was formed by completion of the triple proton transfer. During conversion from 2-TS to 2-PC, significant W1 migration and W2 rotation occurred. 2-PC comprised a capped enolized GI residue and two water molecules. The optimized geometry of 2-PC closely resembled that of 1-PC, and W2 formed three hydrogen bonds with W1 (1.798 Å), the carbonyl carbon oxygen of the (*n* − 1) residue (1.892 Å), and the OH proton of the enol moiety (1.621 Å). During enolization from 2-RC to 2-PC, all the defined dihedral angles changed by less than 30°, and no significant conformational changes occurred in the main and side chains of the GI residue. The relative energies of 2-TS and 2-PC with respect to 2-RC were 35.5 and 20.2 kcal·mol^−1^, respectively.

### 2.3. Pathway 3

**Figure 4:**
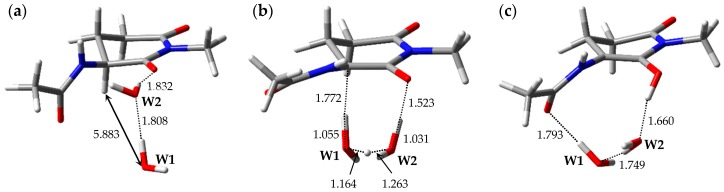
Optimized geometries of the enolization system in Pathway 3: (**a**) 3-RC (*φ* = −120°, *ψ* = −159°, *χ*_1_ = −179°, *χ*_2_ = −52°); (**b**) 3-TS (*φ* = −159°, *ψ* = −160°, *χ*_1_ = −176°, *χ*_2_ = −49°); and (**c**) 3-PC (*φ* = −127°, *ψ* = −170°, *χ*_1_ = −159°, *χ*_2_ = −47°). The selected interatomic distances are shown in Å.

The optimized geometries of the enolization system in Pathway 3, i.e., reactant complex 3-RC, TS 3-TS, and product complex 3-PC, are shown in Figure 4. In the Supplementary Materials, Tables S7–S9 present the Cartesian coordinates of 3-RC, 3-TS, and 3-PC. 3-RC is a complex which is composed of a capped l-GI residue and two water molecules, W1 and W2. Although the conformation of the l-GI residue in 3-RC was similar to those in 1-RC and 2-RC, the arrangement of the catalytic water molecules and interaction modes were remarkably different. In 3-RC, the distance between H_α_ of the l-GI residue and W1 was 5.883 Å. W2 formed two hydrogen bonds, one with W1 (1.808 Å), and the other with the main-chain carbonyl oxygen of the l-GI residue (1.832 Å). As with Pathways 1 and 2, the l-GI-residue enolization proceeded by proton relay via two water molecules. 3-TS is a TS connecting two energy minima, 3-RC and 3-PC, and a single imaginary frequency of 3-TS was estimated as 656*i* cm^−1^ by vibrational frequency calculations. When 3-TS was formed from 3-RC, remarkable migration of catalytic water molecules and a conformational change in the main chain of the l-GI residue occurred (the dihedral angle *φ* was altered by 39°). In 3-TS, the C_α_‒H_α_ bond length was increased to 1.772 Å. Figure S3 shows the transition vector of 3-TS in the Supplementary Materials. Triple proton transfer (along the transition vector) mediated by two water molecules resulted in the formation of 3-PC, which comprised a capped enolized GI residue and two water molecules. When 3-PC was formed from 3-TS, a dramatic main-chain conformational change and water-molecule migration occurred, and a new hydrogen bond was formed between W1 and the carbonyl oxygen of the (*n* − 1) residue (1.793 Å). In 3-PC, W2 interacted with W1 and with the enol OH proton; these hydrogen bond lengths were 1.749 and 1.660 Å, respectively. During enolization from 3-RC to 3-PC, the dihedral angles *ψ*, *χ*_1_, and *χ*_2_ were altered by approximately 20°, whereas the dihedral angles *φ* of 3-RC, 3-TS, and 3-PC were −120°, −159°, and −127°, respectively. A larger change was observed in the dihedral angle *φ*. The relative energies of 3-TS and 3-PC with respect to 3-RC were 34.1 and 17.2 kcal·mol^−1^, respectively.

### 2.4. Pathway 4

**Figure 5:**
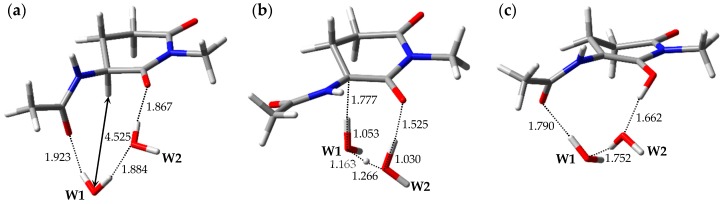
Optimized geometries of the enolization system in Pathway 4: (**a**) 4-RC (*φ* = −106°, *ψ* = −157°, *χ*_1_ = 179°, *χ*_2_ = −52°); (**b**) 4-TS (*φ* = −159°, *ψ* = −160°, *χ*_1_ = −176°, *χ*_2_ = −49°); and (**c**) 4-PC (*φ* = −128°, *ψ* = −170°, *χ*_1_ = −159°, *χ*_2_ = −47°). The selected interatomic distances are shown in Å.

The optimized geometries of the enolization system in Pathway 4, i.e., reactant complex 4-RC, TS 4-TS, and product complex 4-PC, are shown in Figure 5. In the Supplementary Materials, Tables S10–S12 present the Cartesian coordinates of 4-RC, 4-TS, and 4-PC. 4-RC is composed of a capped l-GI residue and two water molecules, W1 and W2, and the distance between W1 and H_α_ of the l-GI residue was 4.525 Å. The dihedral angles *φ*, *ψ*, *χ*_1_, and *χ*_2_ in 4-RC were −106°, −157°, 179°, and −52°, respectively. Although the conformation of the l-GI residue in 4-RC was similar to those in 1-RC and 2-RC, the arrangement and interaction mode of the catalytic water molecules were different. In 4-RC, three hydrogen bonds were formed between W1 and the carbonyl oxygen of the (*n* − 1) residue (1.923 Å), between W1 and W2 (1.884 Å), and between W2 and the main-chain carbonyl oxygen of the l-GI residue (1.867 Å). The l-GI-residue enolization was mediated by triple proton transfer among l-GI and two water molecules. When 4-TS was formed from 4-RC, W1 and W2 migrated significantly, and a hydrogen bond between W1 and the carbonyl oxygen of the (*n* − 1) residue was cleaved. The optimized geometry of 4-TS was similar to that of 3-TS; however, the W2 orientations were significantly different. In 4-TS, the C_α_‒H_α_ bond length was increased to 1.777 Å. 4-TS is a TS connecting two energy minima, 4-RC and 4-PC, and vibrational frequency calculations showed that 4-TS had a single imaginary frequency of 643*i* cm^−1^. Figure S4 shows the transition vector of 4-TS in the Supplementary Materials. When the asynchronous triple proton transfer that occurred along the transition vector with l-GI-residue enolization was complete, 4-PC was formed. 4-PC comprised a capped enolized GI residue and two water molecules. Although the optimized geometry of 4-PC was similar to that of 3-PC, the W2 orientations were significantly different. When 4-PC was formed from 4-TS, a hydrogen bond was again formed between W1 and the carbonyl oxygen of the (*n* − 1) residue, with a hydrogen bond length of 1.790 Å. W2 formed two hydrogen bonds with W1 (1.752 Å) and with the enol OH proton (1.662 Å). During enolization from 4-RC to 4-PC, the changes in the dihedral angles *φ* of 4-RC, 4-TS, and 4-PC were −106°, −159°, and −128°, respectively, and the main-chain conformation of the GI residue changed more than in any other pathway. The relative energies of 4-TS and 4-PC with respect to 4-RC were 35.8 and 18.7 kcal·mol^−1^, respectively.

## 3. Discussion

In the present study, we have successfully identified four enolization pathways from l-GI to enolized GI residues. In all the pathways, enolization proceeded via one step, and two water molecules placed around the capped l-GI residue acted as catalysts. During enolization, triple proton transfer mediated by catalytic water molecules occurred; the protons were transferred from C_α_ of the l-GI residue to W1, from W1 to W2, and from W2 to the carbonyl oxygen of the l-GI residue. The relative energies of all optimized geometries are shown in Table 1, and the total energies and ZPEs are shown in Table S13 in the Supplementary Material. In Pathways 1, 2, 3, and 4, the local activation energies are 35.1, 35.5, 34.1, and 35.8 kcal·mol^−1^, respectively. Among four reactant complexes, 4-RC had the lowest calculated energy, and the calculated energies of 1-RC, 2-RC, and 3-RC with respect to 4-RC were 0.652, 0.655, and 1.53 kcal·mol^−1^, respectively. Thus, the obtained activation energies with respect to 4-RC were estimated as 35.7, 36.1, and 35.6 kcal·mol^−1^ in Pathways 1, 2, and 3, respectively, and all the activation energies obtained did not differ significantly. These computational results suggest that the conformations of l-GI residues and the arrangements of catalytic water molecules are not unique when water molecules catalyze l-GI-residue stereoinversion. However, the conformational changes in the l-GI-residue main and side chains in Pathways 3 and 4 were larger than those in Pathways 1 and 2. Thus, it is suggested that the l-GI-residue main chain must be more flexible to allow enolization via Pathways 3 and 4. In any case, all the activation energies are too high to allow the non-enzymatic reaction to proceed easily under physiological conditions.

Although we obtained the enolization pathways of l-GI residues in the present study, note that the d-GI residue must be formed by reketonization of the enolized GI residue to allow completion of GI-residue stereoinversion. For d-GI residue formation, catalytic water molecules must be arranged on the opposite side to where H_α_ was originally bound, and a proton recombines with C_α_ of the enolized GI residue on the side where the catalytic water molecules are arranged. However, there are abundant water molecules in the living body, and it is conceivable that the catalytic water molecules could be easily arranged on the “opposite side” in the absence of steric hindrance. The d-GI residues are formed by reketonization of the enolized GI residue. Because enolized GI-residue reketonization is the reverse reaction of GI-residue enolization (Scheme 4), the TSs of reketonization and enolization are the same. The reketonization pathways from enolized GI to d-GI residues are mirror images of reketonization from enolized GI to l-GI residues, and the TSs of reketonization are reported in this paper (1-TS, 2-TS, 3-TS, and 4-TS). Thus, the activation energies for the whole reaction from the l-GI to d-GI residues are equal to the energies of 1-TS, 2-TS, 3-TS, and 4-TS. All the activation energies of the l-GI-residue stereoinversion in the aqueous phase obtained in the present study were substantially higher than that of Asp-residue stereoinversion, which was experimentally determined as approximately 25.7–29.0 kcal·mol^−1^ [21].

## 4. Material and Methods

Figure 1 shows the model compound in the present study (Ace‒l-GI‒Me). All calculations were performed using Gaussian 16 software [33]. As in previous studies [28], all the energy optimizations were performed without any constraints by DFT calculations using the B3LYP exchange-correlation functional and 6-31+G(d,p) basis set, and energy minima and TS geometries were obtained. Recently, we performed benchmark studies on the mechanism of nonenzymatic Asp-residue cyclization, and showed that the calculations using B3LYP functional methods provided favorable results [34]. In addition, the activation energies for non-enzymatic modifications of amino acid residues were successfully calculated using the B3LYP/6-31+G(d,p) level of theory [23,24,25,26,27,28,29,30,35,36]. Thus, we used B3LYP/6-31+G(d,p) for this study. Vibrational frequency calculations were performed for all the optimized geometries to confirm them as energy minima (with no imaginary frequency) or TSs (with a single imaginary frequency), and to obtain their ZPEs. Furthermore, intrinsic reaction coordinate calculations were conducted for all the TSs to confirm that each TS connected to energy minima. For all the calculations, the PCM was employed to reproduce the aqueous environment. The dielectric constant of water for the PCM was set to 78.355 (default setting in Gaussian 16). All the relative energies of the energy minima and TS geometries were calculated at the B3LYP/6-31+G(d,p) level of theory and corrected for the ZPEs.

## 5. Conclusions

We have explored the enolization mechanisms of the l-GI residue catalyzed by water molecules in the aqueous phase and successfully identified four reaction pathways. In all the pathways, two water molecules placed around the capped l-GI residue catalyzed proton transfer. The approximate activation energies calculated for all pathways was 36 kcal·mol^−1^. These obtained activation energies were considerably higher than the activation energy of Asp-residue stereoinversion (25.7–29.0 kcal·mol^−1^), as experimentally reported [21]. Thus, the high activation energies of l-Glu-residue stereoinversion may be a reason why stereoinverted and isomerized Glu residues have not been experimentally detected in human proteins.

## Figures and Tables

**Table 1:** Relative energies of optimized geometries, which are shown in kcal·mol^−1^ and were corrected for zero-point energies after being calculated at the B3LYP/6-31+G(d,p) level of theory.

Pathway	Geometry	Relative Energy
1	1-RC	0.641
	1-TS	35.7
	1-PC	21.0
2	2-RC	0.655
	2-TS	36.1
	2-PC	20.9
3	3-RC	1.53
	3-TS	35.6
	3-PC	18.8
4	4-RC	0
	4-TS	35.8
	4-PC	18.7

## Supplementary Materials

Supplementary Materials can be found at https://www.mdpi.com/1422-0067/20/10/2410/s1.

## References

[1] Fujii N., Saito T. (2004). Homochirality and life. Chem. Rec..

[2] Fujii N. (2002). D-Amino acids in living higher organisms. Orig. Life Evol. Biosph..

[3] Ritz-Timme S., Collins M.J. (2002). Racemization of aspartic acid in human proteins. Ageing Res. Rev..

[4] Fujii N., Takata T., Fujii N., Aki K., Sakaue H. (2018). D-Amino acids in protein: The mirror of life as a molecular index of aging. Biochim. Biophys. Acta Proteins Proteom..

[5] Kubo T., Nishimura S., Kumagae Y., Kaneko I. (2002). In vivo conversion of racemized β-amyloid ([d-Ser^26^]Aβ1–40) to truncated and toxic fragments ([d-Ser^26^]Aβ25–35/40) and fragment presence in the brains of Alzheimer’s patients. J. Neurosci. Res..

[6] Shapira R., Chou C.-H.J. (1987). Differential racemization of aspartate and serine in human myelin basic protein. Biochem. Biophys. Res. Commun..

[7] Shapira R., Austin G.E., Mirra S.S. (1988). Neuritic plaque amyloid in Alzheimer’s disease is highly racemized. J. Neurochem..

[8] Roher A.E., Lowenson J.D., Clarke S., Wolkow C., Wang R., Cotter R.J., Reardon I.M., Zürcher-Neely H.A., Heinrikson R.L., Ball M.J. (1993). Structural alterations in the peptide backbone of β-amyloid core protein may account for its deposition and stability in Alzheimer’s disease. J. Biol. Chem..

[9] Fujii N., Satoh K., Harada K., Ishibashi Y. (1994). Simultaneous stereoinversion and isomerization at specific aspartic acid residues in αA-crystallin from human lens. J. Biochem..

[10] Fujii N., Ishibashi Y., Satoh K., Fujino M., Harada K. (1994). Simultaneous racemization and isomerization at specific aspartic acid residues in αB-crystallin from the aged human lens. Biochim. Biophys. Acta Struct. Mol. Enzym..

[11] Kaji Y., Oshika T., Takazawa Y., Fukayama M., Takata T., Fujii N. (2007). Localization of D-β-aspartic acid–containing proteins in human eyes. Invest. Opthalmol. Vis. Sci..

[12] Hooi M.Y.S., Truscott R.J.W. (2011). Racemisation and human cataract. d-Ser, d-Asp/Asn and d-Thr are higher in the lifelong proteins of cataract lenses than in age-matched normal lenses. AGE.

[13] Inoue K., Hosaka D., Mochizuki N., Akatsu H., Tsutsumiuchi K., Hashizume Y., Matsukawa N., Yamamoto T., Toyo’oka T. (2014). Simultaneous determination of post-translational racemization and isomerization of N-terminal amyloid-β in Alzheimer’s brain tissues by covalent chiral derivatized ultraperformance liquid chromatography tandem mass spectrometry. Anal. Chem..

[14] Powell J.T., Vine N., Crossman M. (1992). On the accumulation of d-aspartate in elastin and other proteins of the ageing aorta. Atherosclerosis.

[15] Ritz-Timme S., Laumeier I., Collins M. (2003). Age estimation based on aspartic acid racemization in elastin from the yellow ligaments. Int. J. Legal Med..

[16] Ritz-Timme S., Laumeier I., Collins M.J. (2003). Aspartic acid racemization: Evidence for marked longevity of elastin in human skin. Brit. J. Derm..

[17] Fujii N., Fujii N., Kida M., Kinouchi T. (2010). Influence of Lβ-, Dα- and Dβ-Asp isomers of the Asp-76 residue on the properties of αA-crystallin 70–88 peptide. Amino Acids.

[18] Oda A., Kobayashi K., Takahashi O. (2010). Molecular-dynamics simulations for amyloid β_1–42_ Monomer with D-aspartic acid residues using continuous solvent. Chem. Biodivers..

[19] Sakaue H., Kinouchi T., Fujii N., Takata T., Fujii N. (2017). Isomeric replacement of a single aspartic acid induces a marked change in protein function: The example of ribonuclease A. ACS Omega.

[20] Radkiewicz J.L., Zipse H., Clarke S., Houk K.N. (1996). Accelerated racemization of aspartic acid and asparagine residues via succinimide intermediates:  an ab initio theoretical exploration of mechanism. J. Am. Chem. Soc..

[21] Kuge K., Fujii N., Miura Y., Tajima S., Saito T. (2004). Kinetic study of racemization of aspartyl residues in synthetic elastin peptides. Amino Acids.

[22] Catak S., Monard G., Aviyente V., Ruiz-López M.F. (2009). Deamidation of asparagine residues: Direct hydrolysis versus succinimide-mediated deamidation mechanisms. J. Phys. Chem. A.

[23] Takahashi O., Kobayashi K., Oda A. (2010). Modeling the enolization of succinimide derivatives, a key step of racemization of aspartic acid residues: Importance of a two-H_2_O mechanism. Chem. Biodivers..

[24] Takahashi O. (2013). Two-water-assisted racemization of the succinimide intermediate formed in proteins. A computational model study. Health.

[25] Takahashi O., Oda A., Jones J.E., Morano D.M. (2012). Amide-iminol tautomerization of the C-terminal peptide groups of aspartic acid residues: Two-water-assisted mechanism, cyclization from the iminol tautomer leading to the tetrahedral intermediate of succinimide formation, and implication to peptide group hydrogen exchange. Tyrosine and Aspartic Acid: Properties, Source and Health Benefits.

[26] Takahashi O., Kirikoshi R. (2014). Intramolecular cyclization of aspartic acid residues assisted by three water molecules: A density functional theory study. Comput. Sci. Disc..

[27] Takahashi O., Kirikoshi R., Manabe N. (2014). Roles of intramolecular and intermolecular hydrogen bonding in a three-water-assisted mechanism of succinimide formation from aspartic acid residues. Molecules.

[28] Fukuyoshi S., Nakayoshi T., Takahashi O., Oda A. (2017). Theoretical study on keto-enol tautomerisation of glutarimide for exploration of the isomerization reaction pathway of glutamic acid in proteins using density functional theory. Mol. Phys..

[29] Nakayoshi T., Kato K., Fukuyoshi S., Takahashi O., Kurimoto E., Oda A. (2018). Computational studies on cyclic imide formation mechanism of glutamic acid residues catalyzed by two water molecules. J. Phys.: Conf. Ser..

[30] Nakayoshi T., Kato K., Fukuyoshi S., Takahashi O., Kurimoto E., Oda A. (2018). Computational studies on the water-catalyzed stereoinversion mechanism of glutamic acid residues in peptides and proteins. Chirality.

[31] Foresman J.B., Keith T.A., Wiberg K.B., Snoonian J., Frisch M.J. (1996). Solvent effects. 5. Influence of cavity shape, truncation of electrostatics, and electron correlation on ab initio reaction field calculations. J. Phys. Chem..

[32] Takenaka N., Koyano Y., Nakagawa Y., Nagaoka M. (2009). An optimum strategy for solution chemistry using semiempirical molecular orbital method: Importance of description of charge distribution. J. Comput. Chem..

[33] Frisch M.J., Trucks G.W., Schlegel H.B., Scuseria G.E., Robb M.A., Cheeseman J.R., Scalmani G., Barone V., Petersson G.A., Nakatsuji H. (2016). Gaussian 16, Revision A.03.

[34] Fukuyoshi S., Nakayoshi T., Takahashi O., Oda A. Evaluations of density functionals and solvent effect for racemization reaction of amino acid residues.

[35] Kato K., Nakayoshi T., Kurimoto E., Oda A. (2019). Computational studies on the nonenzymatic deamidation mechanisms of glutamine residues. ACS Omega.

[36] Nakayoshi T., Kato K., Kurimoto E., Oda A. (2019). Possible mechanisms of nonenzymatic formation of dehydroalanine residue catalyzed by dihydrogen phosphate ion. J. Phys. Chem. B.

