# Supplementary material for: Computational Studies on Water-Catalyzed Mechanisms for Stereoinversion of Glutarimide Intermediates Formed from Glutamic Acid Residues in Aqueous Phase
Source: Int J Mol Sci. 2019 May 15;20(10):2410. doi: 10.3390/ijms20102410 (PMC6567045; doi:10.3390/ijms20102410)
Supplement: Supplementary file 1 [file ijms-20-02410-s001.pdf]

Table S1. Cartesian coordinates of 1-RC.

Atom	*x*	*y*	*z*
C	0.000058	0.000416	0.000263
H	−0.001498	0.000089	1.093765
H	1.031809	0.001432	−0.357009
H	−0.492988	−0.917261	−0.332984
C	−0.804000	1.184595	−0.485760
O	−1.988893	1.333008	−0.135362
N	−0.159180	2.057821	−1.293595
H	0.790075	1.838129	−1.565728
C	−0.766220	3.235036	−1.879207
H	−1.713296	3.401473	−1.355117
C	0.108801	4.479717	−1.723796
H	0.289183	4.665863	−0.662050
H	1.082198	4.318653	−2.202516
C	−0.615321	5.667192	−2.354651
H	0.003732	6.566847	−2.362441
H	−1.524745	5.895214	−1.779803
C	−1.054847	5.418025	−3.777147
O	−1.228243	6.328325	−4.580056
C	−1.136326	2.976522	−3.350958
O	−1.313331	1.843231	−3.774831
N	−1.294123	4.094163	−4.175267
C	−1.725826	3.891680	−5.569385
H	−1.930189	2.834205	−5.710830
H	−0.937503	4.214759	−6.252322
H	−2.623524	4.481115	−5.760731
O	−3.645207	5.608284	−0.675782
H	−3.947890	6.089823	0.104760
H	−3.893567	4.669717	−0.522289
O	−4.238507	2.911506	−0.316846
H	−3.439614	2.338210	−0.300105
H	−4.830852	2.529874	−0.978105

Table S2. Cartesian coordinates of 1-TS.

Atom	x	y	z
C	0.000055	−0.000052	−0.000187
H	−0.000358	−0.000472	1.093282
H	1.031172	0.000299	−0.358804
H	−0.492309	−0.919294	−0.331258
C	−0.805790	1.185865	−0.480547
O	−1.978952	1.324785	−0.041184
N	−0.201188	2.010368	−1.345004
H	0.736037	1.717224	−1.605932
C	−0.627759	3.267578	−1.950117
H	−1.771061	4.046063	−1.133063
C	0.543534	4.237581	−2.003839
H	0.890611	4.468752	−0.991339
H	1.402760	3.793939	−2.536894
C	0.127577	5.529535	−2.703211
H	0.981569	6.179529	−2.906295
H	−0.564806	6.097038	−2.066305
C	−0.593773	5.304084	−4.011504
O	−0.587191	6.144856	−4.913186
C	−1.378420	3.097687	−3.174143
O	−2.150534	2.133133	−3.389303
N	−1.331495	4.138426	−4.147474
C	−2.136051	4.007841	−5.371454
H	−2.655231	3.054656	−5.334172
H	−1.485340	4.048337	−6.247388
H	−2.857144	4.826621	−5.434599
O	−2.702551	4.522268	−0.756867
H	−2.614629	4.706450	0.190833
H	−3.454874	3.503182	−0.992130
O	−3.846382	2.452407	−1.250156
H	−3.119707	1.912352	−0.730914
H	−3.578673	2.291034	−2.187259

Table S3. Cartesian coordinates of 1-PC.

Atom	*x*	*y*	*z*
C	−0.000299	−0.000025	−0.000131
H	−0.001333	−0.000958	1.094249
H	1.033490	0.000504	−0.352323
H	−0.494703	−0.916593	−0.332681
C	−0.800332	1.191079	−0.479330
O	−2.017240	1.289776	−0.234709
N	−0.124505	2.123411	−1.197471
H	0.863968	1.955918	−1.348940
C	−0.629955	3.405442	−1.577466
H	−5.866297	2.085863	−3.612220
C	−0.496699	4.548719	−0.604324
H	−0.651859	4.188107	0.417448
H	0.521633	4.966752	−0.637928
C	−1.530549	5.632535	−0.921811
H	−1.333556	6.558154	−0.377876
H	−2.530077	5.283936	−0.625697
C	−1.619763	5.976860	−2.397011
O	−1.894549	7.117921	−2.785160
C	−1.102788	3.622426	−2.823241
O	−1.254071	2.682110	−3.779800
N	−1.439563	4.929629	−3.273582
C	−1.614792	5.148341	−4.715748
H	−0.744517	4.778860	−5.261115
H	−1.720827	6.219312	−4.872030
H	−2.506405	4.633225	−5.080486
O	−5.446995	1.242364	−3.833037
H	−6.103817	0.563951	−3.622848
H	−3.800567	0.982418	−3.161871
O	−2.874605	0.828785	−2.871452
H	−2.847068	0.946397	−1.900679
H	−1.826408	1.926133	−3.441999

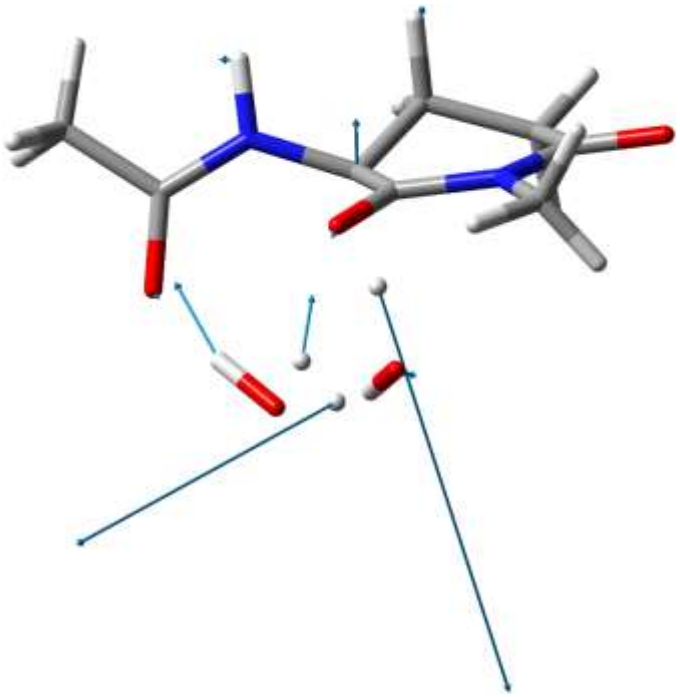

Figure S1. Transition vector of 1-TS.

Table S4. Cartesian coordinates of 2-RC.

Atom	*x*	*y*	*z*
C	−0.000163	−0.000065	0.000145
H	−0.000481	−0.000071	1.093886
H	1.041768	−0.000085	−0.331727
H	−0.490432	−0.907877	−0.357144
C	−0.663128	1.269578	−0.483066
O	−0.196960	2.382951	−0.179961
N	−1.759888	1.116438	−1.260709
H	−2.097725	0.176716	−1.422689
C	−2.543072	2.209732	−1.798310
H	−1.950825	3.121785	−1.669416
C	−2.855432	2.030454	−3.284423
H	−1.920930	1.965724	−3.847511
H	−3.404896	1.095038	−3.444526
C	−3.676183	3.228384	−3.757597
H	−4.007188	3.113461	−4.791882
H	−3.064691	4.141016	−3.705028
C	−4.910093	3.490257	−2.926199
O	−5.887094	4.072546	−3.382587
C	−3.824757	2.413997	−0.973557
O	−3.909880	2.048153	0.190869
N	−4.895270	3.065485	−1.585860
C	−6.082048	3.304810	−0.746522
H	−6.499766	2.352224	−0.415291
H	−6.810612	3.846630	−1.342968
H	−5.799131	3.891529	0.129114
O	−1.646970	5.874680	−2.818178
H	−0.994329	6.382303	−3.317298
H	−1.212533	5.647516	−1.966217
O	−0.493531	5.112012	−0.401413
H	−0.430253	4.136122	−0.297172
H	−0.941921	5.439461	0.389474

Table S5. Cartesian coordinates of 2-TS.

Atom	*x*	*y*	*z*
C	0.000008	−0.000029	0.000054
H	0.002642	0.001046	1.094170
H	1.041526	−0.001101	−0.332862
H	−0.490815	−0.908812	−0.353484
C	−0.659043	1.270576	−0.487868
O	−0.104873	2.369302	−0.215900
N	−1.794067	1.127168	−1.183617
H	−2.100116	0.162530	−1.274617
C	−2.652727	2.093764	−1.862152
H	−1.904275	3.455827	−2.264612
C	−3.162768	1.494520	−3.164743
H	−2.327308	1.293252	−3.843291
H	−3.662501	0.526283	−2.985881
C	−4.144480	2.447294	−3.841885
H	−4.657637	1.974399	−4.682117
H	−3.611070	3.317554	−4.249051
C	−5.208613	2.998500	−2.918441
O	−6.299571	3.373687	−3.350796
C	−3.655831	2.682735	−1.001591
O	−3.481511	2.930603	0.214906
N	−4.879750	3.123750	−1.573968
C	−5.832585	3.782860	−0.667445
H	−6.095915	3.112115	0.151977
H	−6.719493	4.035263	−1.242589
H	−5.387135	4.688206	−0.249727
O	−1.405777	4.449081	−2.363827
H	−1.975907	5.051355	−2.865150
H	−1.360792	4.694625	−1.097726
O	−1.272513	4.555639	0.038395
H	−0.741533	3.656575	0.012098
H	−2.161813	4.248942	0.339472

Table S6. Cartesian coordinates of 2-PC.

Atom	*x*	*y*	*z*
C	−0.000080	−0.000388	−0.000129
H	−0.000720	−0.000750	1.093084
H	1.043031	−0.000196	−0.331173
H	−0.485186	−0.909655	−0.361610
C	−0.659201	1.269608	−0.491138
O	−0.277278	2.387187	−0.096814
N	−1.689440	1.117986	−1.361431
H	−1.930926	0.168668	−1.623256
C	−2.320598	2.171887	−2.092443
H	−3.817575	5.917357	2.127962
C	−1.717907	2.597021	−3.406935
H	−0.625669	2.561445	−3.344669
H	−2.009098	1.899566	−4.207963
C	−2.159780	4.021920	−3.751521
H	−1.917100	4.287883	−4.781962
H	−1.639750	4.735571	−3.096906
C	−3.644246	4.265171	−3.551831
O	−4.278853	5.049336	−4.266351
C	−3.496845	2.692041	−1.682091
O	−4.135106	2.379280	−0.534445
N	−4.226027	3.609718	−2.489046
C	−5.633027	3.868968	−2.155224
H	−6.179727	2.928000	−2.071678
H	−6.052898	4.469606	−2.958711
H	−5.713860	4.410848	−1.210116
O	−3.081843	5.441151	2.537816
H	−3.253485	5.468435	3.489524
H	−2.725181	3.819638	1.848813
O	−2.541880	2.928788	1.477177
H	−1.652338	2.957584	1.070916
H	−3.528762	2.524889	0.255710

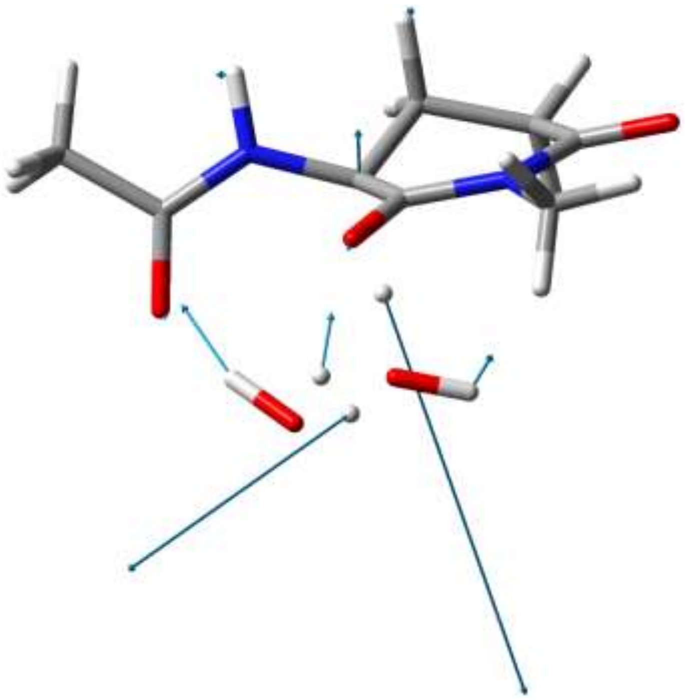

Figure S2. Transition vector of 2-TS.

Table S7. Cartesian coordinates of 3-RC.

Atom	*x*	*y*	*z*
C	−0.001373	−0.000472	0.001021
H	−0.000932	−0.001044	1.094172
H	1.040929	−0.000173	−0.331947
H	−0.487380	−0.909972	−0.359105
C	−0.664524	1.270074	−0.486303
O	−0.292860	2.386559	−0.102602
N	−1.682836	1.108818	−1.377645
H	−1.930295	0.171872	−1.666749
C	−2.361055	2.223051	−2.002616
H	−1.947500	3.125978	−1.534832
C	−2.132736	2.294359	−3.515433
H	−1.061485	2.373502	−3.715837
H	−2.492309	1.375236	−3.992666
C	−2.867898	3.509717	−4.074078
H	−2.830661	3.545386	−5.164917
H	−2.401764	4.435677	−3.710157
C	−4.326587	3.576383	−3.682287
O	−5.147141	4.195368	−4.346062
C	−3.855363	2.215843	−1.666491
O	−4.306492	1.645677	−0.676064
N	−4.718428	2.911025	−2.504055
C	−6.138438	2.940533	−2.108307
H	−6.675834	3.540994	−2.836557
H	−6.232183	3.379411	−1.113710
H	−6.536473	1.924567	−2.088498
O	−3.508254	1.571071	3.920606
H	−3.149345	−0.614157	1.448277
H	−4.303541	1.411509	4.444602
O	−3.807321	0.080503	1.581345
H	−3.623096	1.043640	3.100223
H	−3.836246	0.595647	0.749088

Table S8. Cartesian coordinates of 3-TS.

Atom	*x*	*y*	*z*
C	−0.000050	0.000003	0.000030
H	−0.000141	−0.000006	1.093706
H	1.043418	0.000003	−0.330691
H	−0.479880	−0.914183	−0.358317
C	−0.674092	1.268549	−0.489327
O	−0.308658	2.387817	−0.089164
N	−1.671237	1.099021	−1.391853
H	−1.945830	0.149167	−1.616061
C	−2.422068	2.153018	−2.023741
H	−3.129559	2.835735	−0.549303
C	−1.645701	3.169534	−2.832753
H	−0.864843	3.631458	−2.224723
H	−1.143012	2.680260	−3.683845
C	−2.602967	4.245898	−3.343331
H	−2.123352	4.919621	−4.056684
H	−2.951428	4.862126	−2.501449
C	−3.847913	3.695670	−4.005702
O	−4.442874	4.323937	−4.886843
C	−3.663365	1.721811	−2.525180
O	−4.271532	0.665490	−2.133220
N	−4.322415	2.485112	−3.529808
C	−5.601290	1.966336	−4.033963
H	−5.940696	2.633086	−4.822656
H	−6.340706	1.934382	−3.230342
H	−5.468913	0.957393	−4.427526
O	−3.586066	2.903334	0.399252
H	−4.299281	0.077843	0.900506
H	−4.183201	3.669077	0.407878
O	−4.711806	0.767781	0.359974
H	−4.162770	1.896795	0.494104
H	−4.537883	0.567585	−0.636600

Table S9. Cartesian coordinates of 3-PC.

Atom	*x*	*y*	*z*
C	0.000372	−0.001062	−0.000594
H	0.001207	−0.001101	1.092191
H	1.043219	−0.001319	−0.334065
H	−0.483019	−0.910464	−0.364140
C	−0.664054	1.265035	−0.490278
O	−0.336389	2.381023	−0.040697
N	−1.614341	1.120452	−1.446045
H	−1.852525	0.174653	−1.722419
C	−2.223456	2.190885	−2.161249
H	−1.557703	3.259616	0.934124
C	−1.371471	3.101312	−3.005341
H	−0.416282	3.294207	−2.510573
H	−1.141576	2.619094	−3.968306
C	−2.102138	4.427778	−3.231128
H	−1.610178	5.042425	−3.987133
H	−2.112816	5.003445	−2.294612
C	−3.551253	4.259597	−3.648718
O	−4.109400	5.056271	−4.411692
C	−3.570003	2.284548	−2.211408
O	−4.438136	1.499202	−1.536981
N	−4.222487	3.198742	−3.081323
C	−5.654866	3.013359	−3.349097
H	−5.929169	3.702900	−4.144022
H	−6.247314	3.227227	−2.456313
H	−5.848386	1.986997	−3.665581
O	−2.283233	3.494910	1.556173
H	−3.648989	0.640122	1.486727
H	−2.615157	4.358238	1.275203
O	−4.014538	1.433889	1.073588
H	−3.415583	2.182129	1.322337
H	−4.172182	1.391587	−0.578360

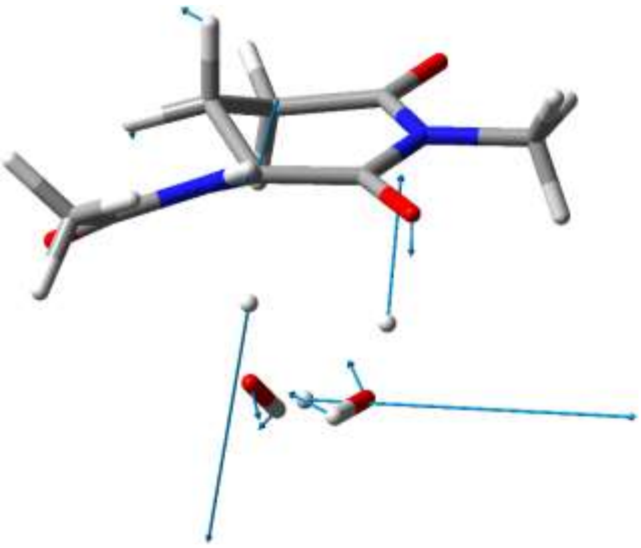

Figure S3. Transition vector of 3-TS.

Table S10. Cartesian coordinates of 4-RC.

Atom	*x*	*y*	*z*
C	−0.000146	0.000047	0.000188
H	−0.000803	−0.000114	1.093795
H	1.042194	0.000253	−0.331163
H	−0.489203	−0.908130	−0.357866
C	−0.662310	1.269070	−0.485731
O	−0.235918	2.391209	−0.162508
N	−1.737345	1.115929	−1.298011
H	−2.051149	0.178198	−1.510882
C	−2.473490	2.225428	−1.865733
H	−1.893457	3.127854	−1.635295
C	−2.641049	2.098584	−3.381105
H	−1.656237	2.063128	−3.853191
H	−3.158420	1.162806	−3.624132
C	−3.434758	3.294670	−3.899544
H	−3.676662	3.194482	−4.959689
H	−2.846456	4.216433	−3.794496
C	−4.739250	3.527719	−3.171748
O	−5.672612	4.123637	−3.691835
C	−3.826847	2.403546	−1.162481
O	−4.022452	2.015441	−0.014005
N	−4.844641	3.051771	−1.850040
C	−6.107674	3.256950	−1.117489
H	−6.797982	3.772130	−1.779127
H	−5.921436	3.857552	−0.225397
H	−6.519082	2.291831	−0.817943
O	−0.409883	3.016825	2.637627
H	−3.680860	2.150329	3.166138
H	−0.184960	2.860509	1.701689
O	−2.979270	1.773927	2.618363
H	−1.303100	2.627516	2.726031
H	−3.305512	1.826704	1.699013

Table S11. Cartesian coordinates of 4-TS.

Atom	*x*	*y*	*z*
C	−0.000881	0.001718	0.002534
H	0.000675	0.004624	1.096205
H	1.042044	0.000018	−0.329908
H	−0.482256	−0.912904	−0.352532
C	−0.674768	1.269454	−0.488907
O	−0.307406	2.389581	−0.092795
N	−1.674174	1.097900	−1.388653
H	−1.953869	0.147832	−1.605538
C	−2.428973	2.149663	−2.019499
H	−3.129393	2.821609	−0.531321
C	−1.654887	3.173221	−2.821954
H	−0.872189	3.628653	−2.211609
H	−1.154669	2.690236	−3.678042
C	−2.612540	4.254307	−3.322112
H	−2.136197	4.930435	−4.035531
H	−2.955839	4.867239	−2.475757
C	−3.858139	3.701286	−3.977832
O	−4.470212	4.321296	−4.853810
C	−3.671605	1.715019	−2.513817
O	−4.263060	0.651098	−2.116919
N	−4.334287	2.491487	−3.511670
C	−5.613443	2.021089	−4.059582
H	−6.399404	2.754367	−3.861207
H	−5.857304	1.075201	−3.584678
H	−5.527898	1.887370	−5.140418
O	−3.575229	2.883608	0.421070
H	−5.561185	0.644159	0.640719
H	−4.171318	3.650160	0.435398
O	−4.626542	0.711041	0.394734
H	−4.156146	1.879994	0.514863
H	−4.535371	0.553838	−0.619461

Table S12. Cartesian coordinates of 4-PC.

Atom	*x*	*y*	*z*
C	0.000030	−0.000080	−0.000045
H	−0.000903	−0.000579	1.092714
H	1.043329	0.000434	−0.332138
H	−0.482498	−0.909529	−0.364579
C	−0.664468	1.265763	−0.489920
O	−0.337384	2.381883	−0.039727
N	−1.613055	1.121067	−1.447204
H	−1.852460	0.174816	−1.720954
C	−2.225423	2.189708	−2.162201
H	−1.562808	3.244790	0.938448
C	−1.376878	3.108249	−3.000746
H	−0.426015	3.308915	−2.500780
H	−1.137826	2.629391	−3.963158
C	−2.118124	4.428626	−3.228440
H	−1.627767	5.048652	−3.981077
H	−2.138498	5.002775	−2.291142
C	−3.563674	4.248639	−3.653549
O	−4.124588	5.040879	−4.419056
C	−3.572610	2.272514	−2.218662
O	−4.436546	1.479487	−1.548068
N	−4.229036	3.182052	−3.089897
C	−5.658172	2.984024	−3.365654
H	−6.257131	3.189167	−2.475190
H	−5.840355	1.956979	−3.686780
H	−5.934906	3.673713	−4.159608
O	−2.299236	3.475417	1.549676
H	−4.705115	1.307090	1.565772
H	−2.648705	4.323717	1.245238
O	−3.890445	1.312846	1.045322
H	−3.390426	2.125171	1.311074
H	−4.159214	1.357285	−0.594237

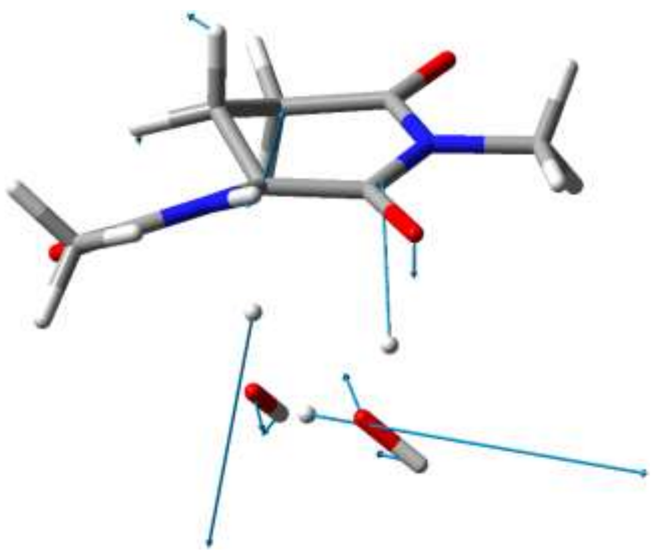

Figure S4. Transition vector of 4-TS.

Table S13. Total energies and zero-point energies of optimized geometries. All the energies are shown in a.u..

Pathway	Geometry	Total energy	Zero-point energy
1	1-RC	−800.268665	0.252307
	1-TS	−800.207008	0.246578
	1-PC	−800.235301	0.251323
2	2-RC	−800.268645	0.252307
	2-TS	−800.206411	0.246578
	2-PC	−800.235419	0.251323
3	3-RC	−800.266844	0.251900
	3-TS	−800.207517	0.246946
	3-PC	−800.239668	0.252202
4	4-RC	−800.270485	0.253104
	4-TS	−800.207502	0.247106
	4-PC	−800.239793	0.252197
